# Towards the concept of disease-modifier in post-stroke or vascular cognitive impairment: a consensus report

**DOI:** 10.1186/s12916-017-0869-6

**Published:** 2017-05-24

**Authors:** Régis Bordet, Ralf Ihl, Amos D. Korczyn, Giuseppe Lanza, Jelka Jansa, Robert Hoerr, Alla Guekht

**Affiliations:** 1 University of Lille, Inserm, CHU, U1171 ‘Degenerative and vascular cognitive disorders’, Lille, France; 2 University of Duesseldorf, Alexian Research Center, Krefeld, Germany; 3 Department of Neurology, Tel Aviv University, Ramat Aviv, Israel; 4 Department of Neurology IC, Oasi Institute for Research on Mental Retardation and Brain Aging (IRCCS), Troina, Italy; 5 University Medical Centre Ljubljana, Neurologic Hospital, Neurorehabilitation Unit, Ljubljana, Slovenia; 6 Dr. Willmar Schwabe GmbH & Co. KG, Karlsruhe, Germany; 7 Department of Neurology, Neurosurgery and Genetics, Russian National Research Medical University, Moscow Research and Clinical Center for Neuropsychiatry, Moscow, Russia; 8 Département de Pharmacologie Médicale, Faculté de Médecine, 1 place Verdun, 59045 Lille Cedex, France

**Keywords:** Disease-modifying therapy, Post-stroke cognitive impairment, Vascular cognitive impairment, Vascular dementia, Clinical trial, Multimodal approach

## Abstract

**Background:**

Vascular cognitive impairment (VCI) is a complex spectrum encompassing post-stroke cognitive impairment (PSCI) and small vessel disease-related cognitive impairment. Despite the growing health, social, and economic burden of VCI, to date, no specific treatment is available, prompting the introduction of the concept of a disease modifier.

**Consensus and suggestions:**

Within this clinical spectrum, VCI and PSCI remain advancing conditions as neurodegenerative diseases with progression of both vascular and degenerative lesions accounting for cognitive decline. Disease-modifying strategies should integrate both pharmacological and non-pharmacological multimodal approaches, with pleiotropic effects targeting (1) endothelial and brain–blood barrier dysfunction; (2) neuronal death and axonal loss; (3) cerebral plasticity and compensatory mechanisms; and (4) degenerative-related protein misfolding. Moreover, pharmacological and non-pharmacological treatment in PSCI or VCI requires valid study designs clearly stating the definition of basic methodological issues, such as the instruments that should be used to measure eventual changes, the biomarker-based stratification of participants to be investigated, and statistical tests, as well as the inclusion and exclusion criteria that should be applied.

**Conclusion:**

A consensus emerged to propose the development of a disease-modifying strategy in VCI and PSCI based on pleiotropic pharmacological and non-pharmacological approaches.

## Background

Given the aging population, increased prevalence of cognitive impairment and dementia in the coming decades has been likened to a “tsunami”, due to the human, social, and economic consequences they will engender [1]. Cerebral or systemic vascular diseases play a relevant role in this “tsunami”. Vascular cognitive impairment (VCI) leading to vascular dementia (VaD) is a clinical spectrum that can be related either to an acute event, namely a stroke (i.e., post-stroke cognitive impairment; PSCI), or to progressive vascular lesions (leukoaraiosis, Binswanger leukoencephalopathy, white matter lesions, microbleeds) related to small vessel disease. Post-stroke VaD and VaD itself were the first presentations to be recognized. VaD was initially represented by multi-infarct dementia, although the concept was then expanded to subcortical ischemic VaD, strategic-infarct dementia, hypoperfusion dementia, and hemorrhagic dementia. In a second step, the existence of mixed Alzheimer’s disease (AD) and VaD was recognized [2]. More recently, cognitive decline has been identified to exist even prior to the stage of overt dementia, and mild cognitive impairment related to vascular lesions has been identified as the preferred target of therapeutic strategies in order to slow or stop the decline, thus avoiding progression towards dementia and related loss of autonomy in daily living.

**Fig. 1:**
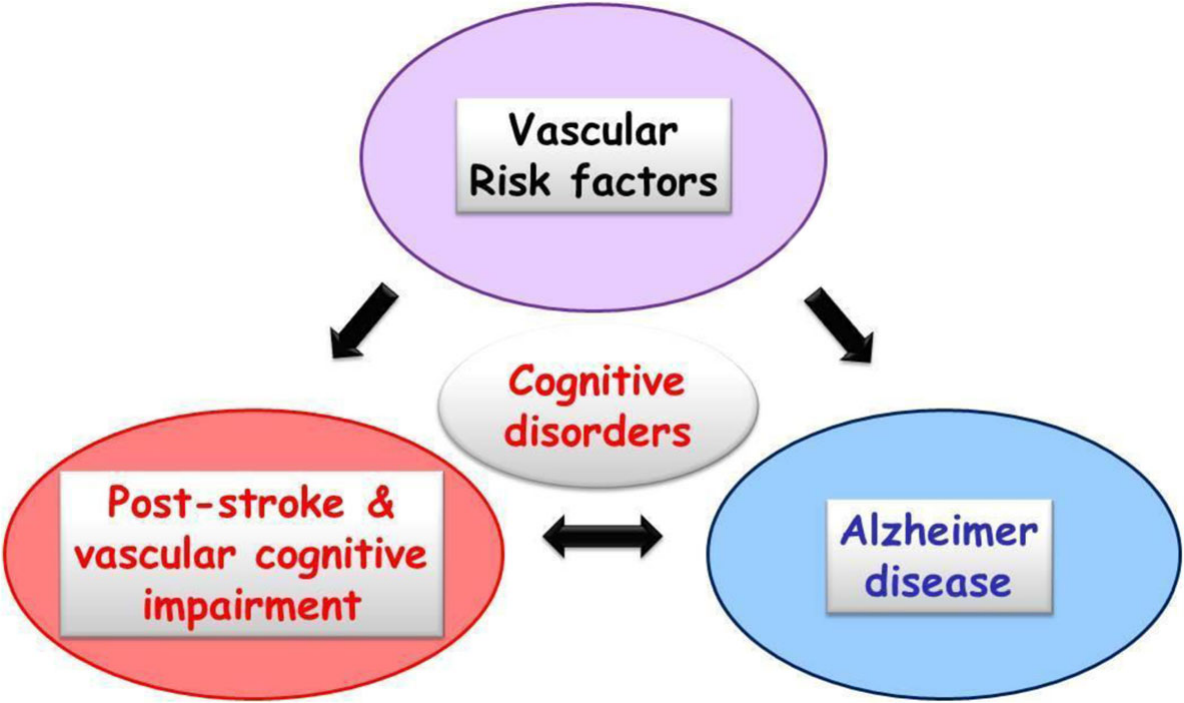
The crossroad of cognitive disorders. Cognitive disorders may be related to vascular or degenerative processes, or both in the mixed form. Vascular risk factors influence both vascular- and degenerative-related cognitive impairment, but also have a direct impact on cognitive functions

**Fig. 2:**
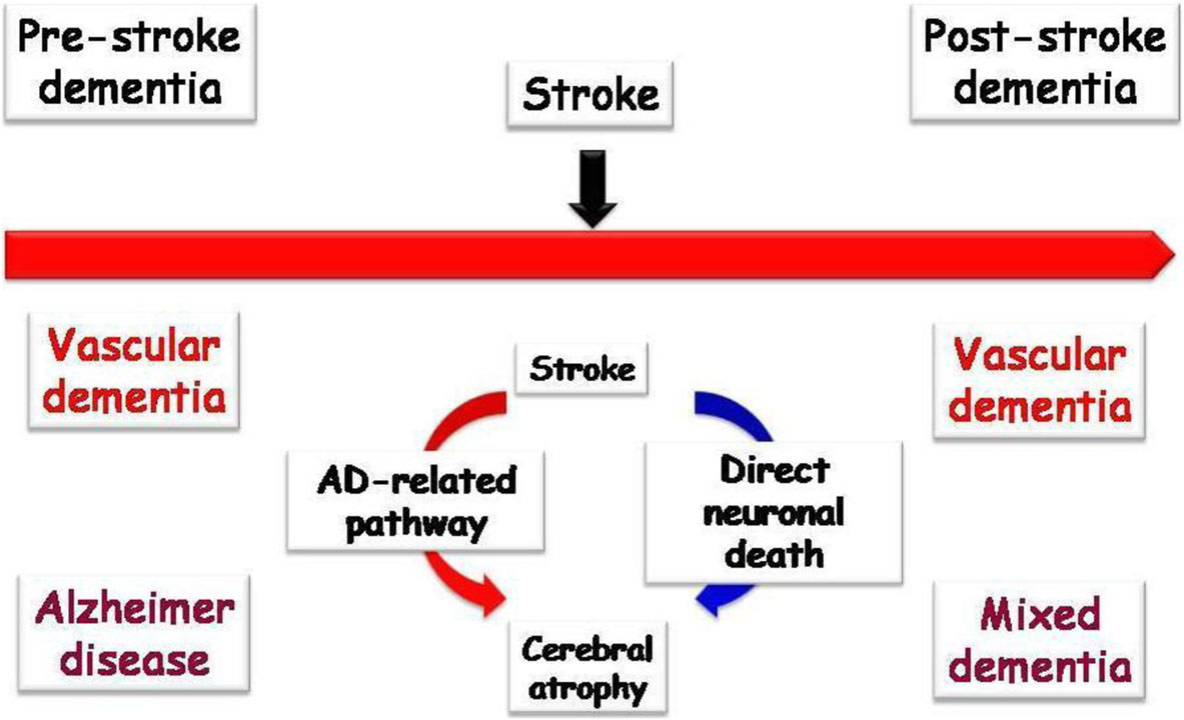
Interactions between pre- and post-stroke cognitive disorders and dementia. Pre-stroke dementia may be related to vascular or degenerative processes, indicating that cognitive impairment after a stroke can be associated with a pure vascular mechanism or a mixed process. Stroke induces regional atrophy through neuronal death directly related to the vascular lesion, or indirectly to the Alzheimer’s disease (AD)-related pathway

All clinical aspects of VCI share the frequent association with vascular risk factors or systemic vascular diseases that lead patients to be prone to developing large or small artery remodeling, explaining the occurrence of vascular brain lesions. The scenario is more complex when considering the potential direct impact of vascular or metabolic risk factors on cognition and the interaction between vascular load and neurodegenerative lesions such as AD-related pathology [3, 4] (Fig. 1). Frailty syndrome could be also a contributor to this complexity [5]. Cognitive dysfunction and dementia frequently occur following an acute stroke, and they are an important cause of stroke-related morbidity. Dementia may be related to a pure VaD or to a mixed form, which occurs after a stroke, or can represent the progression of pre-stroke vascular or degenerative-related cognitive impairment [6] (Fig. 2). There is a large variability in terms of manifestations of cognitive decline in a vascular context, and a delay in the diagnosis when there are no evident or acute symptoms is often observed. The occurrence of an acute event, such as a stroke, provides unique opportunities to follow cognitive function and for the early detection of VCI from a preventive or therapeutic perspective. Indeed, prevention and treatment of PSCI and VCI are the critical priorities for both clinical care and scientific research. The growing health, social, and economic burden of PSCI or VCI is driving the demand for clinical studies that evaluate the benefits and risks of both pharmacological and non-pharmacological interventions [7]. A better understanding of the risk factors and an estimation of the risk scores for PSCI or VCI are of crucial importance for the selection of patients and the design of preventive clinical trials.

Despite the presence of an initial acute event, both VCI and PSCI remain pathodynamic conditions, as neurodegenerative diseases, since there is a progression of both vascular and degenerative lesions accounting for cognitive decline [8]. A prodromal phase might be represented by the period when vascular or metabolic risk factors induce silent brain lesions or by the frailty syndrome, which has been recently considered as a prodromal of VaD [9]. In terms of treatment, research has focused largely on the acute phase of vascular lesions rather than their progression, whereas the main issue remains related to the combination of both vascular and degenerative factors that overlap, despite exhibiting distinctive pathophysiological mechanisms [10]. At the present time, the proposal of the concept of a disease modifier within the spectrum of VaD is warranted. Nevertheless, several issues should be addressed to make this concept operant in its specific field given the complexity of this medical spectrum in terms of evolution (occurrence or not of an acute event), cognitive phenotype, lesion burden, and pathophysiology (pure or mixed lesions). It is also necessary to highlight the differences and similarities with the concept of a disease modifier in the context of neurodegenerative diseases. A disease-modifying strategy, defined as a therapeutic approach aiming to change the natural course of an illness, applies primarily to chronic diseases. In the field of neurological disorders, this concept has been used for neurodegenerative and neuroinflammatory diseases. The mechanisms involved in these conditions are multiple, and therapeutic options are not limited to a single neuroprotection but are integrated in a multimodal approach, including anti-inflammatory, antioxidants or vascular mechanisms, as well as the modulation of protein aggregation and synaptic or neuronal plasticity [11, 12]. Along with neurodegenerative diseases, PSCI and VCI share a preclinical phase prior to the progressive appearance of the first symptoms or after an acute event such as a stroke. PSCI and VCI also resemble neuroinflammatory diseases that show the peculiarity, in their remittent form, to couple the occurrence of acute episodes with a gradual evolution to functional deficits, especially related to motor and cognitive manifestations. Therefore, the natural clinical history suggests that the concept of a disease-modifying effect is applicable to PSCI and VCI, as well as in neurodegenerative or neuroinflammatory pathologies. Moreover, particularly in older patients, VCI or PSCI are rarely “pure” but more often combined with AD-type lesions that contribute to the evolution and justify a disease-modifying strategy [6].

When embarking on trials of potentially disease-modifying treatments for VCI, a look at what we have learnt from disease modification and prevention trials in the neighboring field of AD is commendable. Assuming that the β-amyloid (Aβ) pathology is causally related to dementia in AD, anti-amyloid treatments (e.g., γ-secretase inhibitors, monoclonal antibodies) have been considered as disease-modifying agents par excellence, although a close relationship between amyloid and cognition has not been well established [13]. After a number of failed clinical trials, the role of amyloid is being re-considered, although its feasibility as a therapeutic target has become questionable; even inhibition of γ-secretase led to a faster cognitive and functional decline, probably due to the involvement of this enzyme in other biochemical pathways such as in *notch* signaling [14]. Anti-Aβ immune therapy is still being tried in mild to moderate AD. However, positron emission tomography studies have shown that amyloid deposition mostly takes place before the dementia stage of AD and reaches a plateau once overt dementia is present [15]. Aβ is therefore still considered by some to be the right target, but the dementia stage of the disease may be beyond the window of opportunity. Moreover, amyloid imaging sub-studies of two bapineuzumab phase-III trials found that, in 6.5% of Apolipoprotein E ε4 carriers and in 36% of non-carriers, the cortical amyloid load was below the specified threshold for amyloid positivity (i.e., the therapeutic target was missing) [16]. Similar rates of patients with sparse or lacking amyloid plaques were found in an autopsy trial involving 200 patients with a primary diagnosis of AD [17]. In a number of trials testing the disease-modifying potential of treatments, progression to dementia was chosen as the primary outcome (e.g., [18]). To date, the exact determination of the temporal point at which, during the course of an ongoing disease, a specific threshold is crossed has remained problematic [19].

The multifactorial pathogenesis of PSCI and VCI needs to consider drug combinations or multimodal agents to change the course of the disease, as well as the search of selective ligands targeting distinctive cellular or molecular pathways. Beyond pharmacological agents, non-pharmacological approaches might also be included in this scenario. Nevertheless, potential pitfalls should be taken into consideration, including (1) addressing the wrong target, (2) interfering with the target pathology outside the window of opportunity, (3) patients lacking the target pathology, and (4) choosing insensitive outcomes.

The main objective of this paper is to demonstrate the relevance of a disease-modifying multimodal strategy based on the known pathophysiological pathways and to propose a model of preclinical and clinical development. Even though PSCI forms part of the VCI spectrum, the two entities are distinguished throughout since the different onset type for the two clinical conditions is able to induce a difference in the assessment of the disease-modifying strategy.

## Methodology

A focus meeting on disease modifiers in VCI was held as a part of the “9th International Congress on Vascular Dementia” in Ljubjana, Slovenia, in October 2015. Experts in this field attending the meeting reviewed the current evidence and literature data. For the purpose of this topic review, a comprehensive search on Medline, PubMed, and Embase databases for studies published until April 30, 2016, was conducted. The keywords used in the current search were disease modifier, disease-modifying drug, disease-modifying therapy, disease-modifying strategy, post-stroke cognitive disorder, post-stroke dementia, vascular cognitive impairment, and vascular dementia. Following review of all relevant abstracts, few articles were related to the topic of the paper, prompting the panel experts to define, by themselves, the concept and to highlight the main themes enriching the concept, namely the pathophysiology and pharmacological targets, the potential role of existing drugs, the interest of non-pharmacological approaches, and the need for a specific methodology for future clinical trials. A proposal on disease modifiers in PSCI and VCI was drafted and reviewed by the group. The draft was repeatedly circulated and discussed before being finalized.

### From pathophysiological mechanisms to pharmacological targets for emerging strategies

The association of stroke and dementia is frequent and can be seen either in the diagnostic work-up of patients attending a memory clinic or during the follow-up of stroke survivors [6, 20, 21]. Both ischemic and hemorrhagic strokes lead to a high risk of cognitive impairment and dementia [6, 20, 22]. About one in ten patients has dementia prior to the first stroke, one in ten develops new dementia after the first stroke, and more than one in three develops dementia after a shortly recurrent stroke [23]. A genetic susceptibility has been identified through several gene polymorphisms associated with PSCI or VCI occurrence, in particular the Apolipoprotein E polymorphism [24].

Animal experimental studies on mechanisms and predictors of post-stroke dementia might provide keys for preventive and disease-modifying treatment. Many animal models have been developed to identify some mechanisms of action, including (1) long-term follow-up of middle cerebral artery occlusion; (2) transgenic mice reproducing certain genetic diseases associating stroke and cognitive impairment such as cerebral autosomal dominant arteriopathy with subcortical infarcts and leukoencephalopathy (CADASIL); (3) long-term models of risk factors or bilateral carotid stenosis; and (4) combination of vascular and degenerative lesions in transgenic mice [25, 26]. The assessment of these models combines behavioral tasks, neuroimaging, and cellular and molecular explorations. A recent review emphasizes the role of different animal models in VCI, concluding that no model is able to completely replicate the clinical and pathologic aspects of VCI spectrum explaining, at least in part, the gap between experimental results and translation to clinical reality [27]. In addition, models mimic lesions rather than cognitive decline, with a major limitation in terms of follow-up duration. The development of long-term models should be a priority to improve the relevance of animal experiments. In addition, new approaches should be tested for pharmacological studies in several models to bring more convincing results before a translation to the clinical steps.

International cohort-based consortia (Strokog or Metacohort) are sharing data on biomarkers (genetic, biological, neuroimaging) sampled in stroke patient cohorts with long-term follow-up to assess cognitive evolution [28, 29]. For example, the STROKDEM (Study of Factors Influencing Post-stroke Dementia; NCT01330160) and DEDEMAS (Determinants of Dementia After Stroke) studies are looking at biomarkers (neuroimaging, biological or genetic markers, endothelial function-derived factors) and interactions between vascular and neurodegenerative mechanisms, with an emphasis on the impact of secondary prevention treatments [30]. The Tel-Aviv Brain Acute Stroke Cohort (TABASCO) trial, with up to 10 years follow-up after stroke, is focused on the association between predefined demographic, psychological, inflammatory, biochemical, neuroimaging, and genetic markers measured during the acute phase [31].

**Fig. 3:**
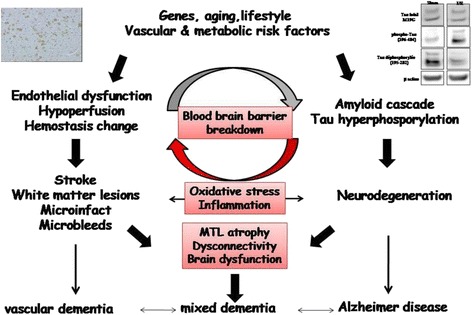
Pathophysiological mechanisms of interactions between vascular and degenerative processes in both vascular and mixed dementia (Adapted from [40]). *MTL* medial temporal lobe. The figure integrates an image of western blot (tau expression after middle cerebral artery occlusion in mice) and immunohistochemistry (amyloid peptide expression after middle cerebral artery occlusion in rat)

The relationship between stroke and cognitive disorders remains complex given that a stroke may induce cognitive impairment through several mechanisms that are often cumulative or synergistic; for example, (1) vascular brain lesions (infarcts or hemorrhages) can be symptomatic by themselves through a location in a strategic area in terms of cognitive functioning; (2) previous silent vascular lesions (leukoaraiosis, Binswanger leukoencephalopathy, microbleeds, silent infarcts, silent hemorrhages, atrophy) may contribute to the burden responsible for cognitive impairment through a cumulative effect or dysconnectivity; (3) accelerated evolution of pre-existing degenerative lesions through hypoxia mechanism; (4) direct effect of vascular or metabolic risk factors associated with stroke occurrence on cognitive functioning; (5) direct induction of neurodegeneration responsible for global or regional brain atrophy; (6) impairment of endothelium function and blood–brain barrier leakage; and (7) induction of neuroinflammation [32, 33] (Fig. 3).

**Fig. 4:**
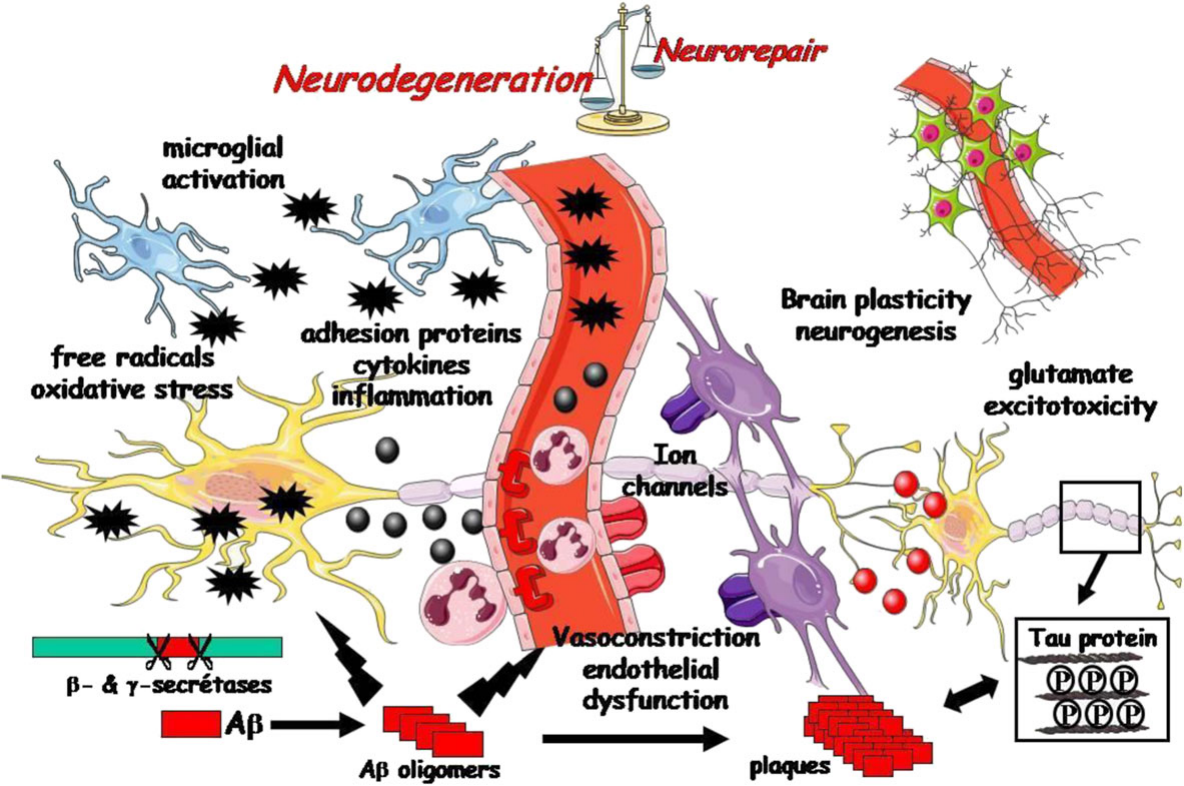
Cellular and molecular mechanisms of vascular dementia and post-stroke cognitive disorders: presentation of all pathways involved in the neurovascular unit and their contribution to vascular impairment and neuronal injury responsible for occurrence and progression of cognitive decline. *Aβ* beta-amyloid

A better understanding of the cellular and molecular processes involved in the dysfunctional neurovascular unit may lead to improved treatments for both PSCI and VCI. Increased attention is being paid to anti-inflammatory strategies, as suggested by the extension and persistence of neuroinflammation after a stroke that may interact with pre-existing AD pathology and accelerate neurodegeneration [34]. Oxidative stress remains an important pathway involved in both neuronal and endothelial injury [35]. Neurovascular uncoupling is also responsible for disturbance of brain oxygenation and vascular reactivity necessary to supply sufficient blood flow in response to neuronal metabolism [36]. Neurohormonal pathways, changes in brain plasticity or neurotrophic factors, ion channels and mitochondrial dysfunction have all been observed in PSCI [37–39]. Stroke is also able to interact with the specific pathophysiology of neurodegenerative pathways (amyloid or *tau* cascades), as vascular or metabolic factors modulate the amyloid or *tau* cascades associated to AD [32, 40]. In contrast, amyloid lesions co-existing with VCI are associated with a greater cognitive dysfunction [41]. The cognitive deterioration is also related to an impairment of neurotransmission pathways, in particular cholinergic or glutamate transmission. More animal and clinical studies are needed to better characterize all the molecular mechanisms contributing to neuronal damage in PSCI and VCI as well as pharmacological targets aiming to develop a disease-modifying therapy with pleiotropic compounds or multimodal combinations targeting (1) endothelial function and blood–brain barrier; (2) neuronal death; (3) cerebral plasticity and compensatory mechanism; or (4) degenerative disease-related protein misfolding (Fig. 4).

### Is there a place for existing drugs?

As demonstrated by the long history of neuroprotection in stroke and the failure of development of new compounds in AD, the search of new drugs remains challenging, thus explaining the ongoing research to find additional properties to drugs currently in use or the development of new indications for existing drugs and fitting with relevant targets for PSCI or VCI [7, 42, 43]. The strategy of drug repurposing would be more successful if the assessed drugs were used in a disease or a risk factor closely related to PSCI or VCI. In this context, the focusing of symptomatic drugs on the treatment of cognitive or behavioral symptoms could also be of interest through additional mechanisms related to disease-modifying effects. Beyond pharmacological options, dietary supplements might be also considered in this regard if they target one or more relevant pathways as, for example, omega-3 [44].

Currently available symptomatic drugs are of interest since evidence-based data in controlled clinical trials have demonstrated the beneficial effect of acetylcholinesterase inhibitors and memantine administration to improve cognition, behavior, and activities of daily living in some patients [45, 46]. Memantine has a lower evidence-based impact but is well tolerated, improves function, and reduces care dependency in treated patients compared to placebo [47]. The size of the effect remains low but is not negligible for patient care to decrease aggressivity or apathy, even though some guidelines do not recommend its general use [42, 47]. Nevertheless, the use of symptomatic drugs as and disease-modifying drugs should not be considered antagonistic, since patients require improvement of a complementary symptomatic effect in addition to the retardation of a disease not immediately detected by the patients or their carers. Inhibitors of acetylcholinesterase or memantine should participate in a multimodal strategy, particularly since they have experimentally demonstrated specific pharmacological effects on certain cellular and molecular pathways involved in PSCI or VCI [39]. The same holds true with antidepressant drugs and omega-3 fatty acid, both of which could act on mood (which is often impaired in such patients) and brain plasticity or neurotrophic factors [48].

The treatment of vascular risk factors offers obvious possibilities for the prevention and impact on disease progression. Obesity, metabolic syndrome, physical inactivity, hypertension, hypercholesterolemia, and other vascular risk factors are associated with cognitive decline and dementia. The relationship between diabetes, stroke, and dementia is also well known [31, 49–52]. Compared with the general population, people with type 2 diabetes have a 1.5- to 2.5-fold increased risk of dementia and, to date, one in 10 to 15 cases of dementia can be attributed to type 2 diabetes [53]. At the cellular level, type 2 diabetes is associated with mitochondrial dysfunction, endoplasmic reticulum stress, increased inflammation, and altered energy metabolism. Anti-diabetic drugs act on hyperglycemia, dyslipidemia, and insulin resistance, and can counteract associated tissue inflammation. A number of anti-diabetic drugs, such as metformin, thiazolidinediones, and compounds targeting the glucagon-like peptide-1 receptor, are able to influence brain metabolism, neuroinflammation, and regeneration. These drugs could be developed as disease-modifying therapies for human brain diseases in patients with and without diabetes [54–56]. The impact of lipid-lowering drugs remains controversial, even though statins and fibrates have demonstrated anti-inflammatory, anti-oxidant, and endothelial properties as well as the ability to modulate brain plasticity through stem cell endogenous mobilization or the AD-related amyloid cascade [7, 57–59]. All anti-hypertensive drug classes have demonstrated similar impact on cellular and molecular pathways. Pharmaco-epidemiological data also suggest that the use of anti-hypertensive drugs is associated with prevention or delayed occurrence of dementia, even though the evidence-based demonstration remains limited to few old clinical trials. Recent meta-analysis pointed out the interest of renin-angiotensin system modulation with both inhibitors of angiotensin converting enzyme and antagonist of angiotensin-2 receptors [60]. The combination of several drug classes could produce a higher effect, with variability of response in function of the etiology of stroke or vascular lesions. This potential favorable impact might be explained by the action of these drugs on the super-family of nuclear receptors, the so-called peroxisome proliferator-activated receptor, which have been demonstrated to exert pleiotropic effects within the brain [61]. Profiling drugs for vascular or metabolic risk factor treatment might be helpful in the selection of more interesting molecules primarily used in the secondary prevention after a stroke.

There is increasing evidence that alterations within the brain neurotrophic support, in particular the brain-derived neurotrophic factor (BDNF) and the nerve growth factor (NGF) expression and signaling, may contribute to neurodegeneration. Indeed, one of the promising strategies of the modifying therapies might be associated with the use of specific neurotrophic factors as well as their modulation by antidepressant or omega-3 fatty acid agents [62, 63]. For instance, cerebrolysin is a peptide preparation with neurotrophic activity demonstrated in different in vitro and in vivo models; its mechanism of action might involve the modulation of the pro-NGF/NGF balance and a concomitant protection of cholinergic neurons [64]. The results of the large, multicenter, double-blind, placebo-controlled study in VaD demonstrated that the drug significantly improved the clinical outcome in VaD patients; moreover, it was safe and well tolerated. The Cochrane review stated that cerebrolysin may have positive effects on both cognitive and global functioning in elderly patients with VaD of mild to moderate severity [65–67]. Actovegin, a deproteinized ultrafiltrate of calf blood comprising more than 200 bioactive constituents, exhibits a range of pleiotropic neuroprotective and metabolic effects [68–70]; a study in a rat model of transient global cerebral ischemia found that it improved spatial learning and memory [70]. The recently completed ARTEMIDA study showed that actovegin (2000 mg i.v. solution for up to 20 daily infusions followed by 1200 mg/day orally for the 6-month remaining period) improved cognitive outcomes in patients with PSCI compared to placebo [71, 72]. Recent pre-clinical studies on *Ginkgo biloba* extract EGb 761 demonstrated that it improves mitochondrial function and energy metabolism, promotes hippocampal neurogenesis and plasticity, and enhances cerebral blood flow by decreasing blood viscosity [73, 74]. This multi-factorial pharmacodynamic profile associated with safety and some symptomatic benefits in clinical studies of dementia [75–77] might provide promising signals for the possible disease-modifying potential that need to be investigated in further clinical trials.

### Is there an evidence of the disease-modifying effect of the non-pharmacological strategies?

Repetitive transcranial magnetic stimulation (rTMS) and transcranial direct current stimulation (tDCS) are non-invasive and painless brain stimulation techniques able not only to explore cortical circuits and related neurochemical pathways in dementing illnesses but also to induce cortical plasticity with potential therapeutic and rehabilitative purposes [78, 79]. Several studies, although methodologically heterogeneous and most of them open-label in design, have shown that specific paradigms of stimulation might improve cognitive performance, thus possibly becoming an alternative to conventional neuroleptic therapy for psychiatric symptoms of dementia [79]. In AD, these effects are probably mediated by compensatory mechanisms supporting the residual abilities [80], and the efficacy can be maximized by selecting patients on the basis of putative neurophysiological markers [78]. Although less is known, similar plastic phenomena are invoked in VaD [81]. High frequency rTMS over the left dorsolateral prefrontal cortex improved executive performance in patients with subcortical ischemic vascular disease, and the effect has been hypothesized to be due to an indirect activation of dopaminergic neurons in the midbrain and the noradrenergic and serotonergic neurons in the brainstem [82]. Moreover, rTMS is also possibly effective in alleviating symptoms of vascular depression [83]. More recently, the restorative effects of these techniques on cognitive ability have been observed in a murine model of VaD, possibly through the neurotrophin release (such as the BDNF) and the induction of hippocampal NMDA-mediated synaptic plasticity [84, 85]. Summarizing, preliminary results revealed that rTMS and tDCS can induce beneficial effects on specific cognitive domains and neuropsychiatric manifestations, although there are limited data and their clinical significance needs to be further validated. Major challenges exist in terms of appropriate patient selection and optimization of the stimulation protocols.

Alternative non-pharmacological strategies identified from traditional medicine should be considered. A systematic review tested the interest of Chinese herbal medicine as an adjunctive and even though some data suggest that this approach could improve cognitive impairment and enhance immediate response and quality of life in VCI patients, limitations of methodological quality warn to improve the design of clinical studies [86, 87]. A recent meta-analysis also suggests a potential effect of acupuncture, but a better definition of trial design is mandatory [88, 89].

Considering the fact that VaD may be associated with a history of stroke with residual motor, perceptual, and cognitive deficits, patients should also have an access to multidisciplinary neurorehabilitation. Further, physiotherapy and occupational therapy (OT) should be based on the up-to-date evidence and consider the impact of cognitive problems on walking ability, falls, and daily functioning [90]. Physical activity may have a significant positive effect on cognitive functioning and ability to perform activities of daily living in the elderly, including patients with both degenerative and vascular dementia [91]. Both the individual and group physical interventions of different types, such as walking, gait and balance training, endurance training, and aerobic-based exercises, have been promising to delay the progression of cognitive decline and to improve physical and mental health [92, 93]. The exercise routine, however, should be based on patient preferences and adjusted to their capabilities. The emerging evidence coming from neurorobotics and exergaming technology (video games) also provide encouraging results, especially because they enhance the adherence to rehabilitation interventions. Accelerometers are becoming an important method of physical assessment as they can be used in real life situations.

Recent evidence coming from Parkinson’s disease and healthy aging population studies suggests that engagement in non-exercise physical activity (including involvement in any meaningful occupation) may also have an independent effect on functional performance [94, 95]. VaD typically leads to impairments in the performance of daily occupations, a key focus of the OT intervention process, and therefore patients with VaD are commonly referred to OT. Within the OT process, the activities of daily living, patient life roles, participation in enjoyed hobbies, and social, recreational, and leisure activities are explored [96]. The underlying reasoning for an effective performance of patients with VaD could be based on the Person-Environment-Occupation model, relying on the degree of fit between person, environment, and occupation. Occupational challenges occur when there is a poor congruence among a person’s capacity, demands of the chosen occupation, and environmental supports and barriers [97]. Assessment results, based on the observation of actual occupational performance, have been shown to be a better indicator of a patient’s ability to resume independent living than assessment of the level of impairment alone (such as the assessment of executive function, apraxia, apathy, and others) [98]. Moreover, it has been shown that OT improved daily functioning of people with dementia and reduced the burden on the caregiver, despite the patient’s limited learning ability [99]. In addition, it has been reported that the quality of the relationship between the person with dementia and their caregiver is an important predictor of whether a patient with dementia will stay in the community or need to enter an institution for ongoing care [100]. To promote well-being, patient choices will be promoted and encouraged, and new hobbies introduced, relevant to an individual’s living context. Examples of leisure activities may include horticultural therapy, regular social and recreational activities, *Tai Chi* [101], music therapy [102], and art therapy. However, further evidence for their efficacy is needed.

In conclusion, PSCI and VCI patients might benefit from ongoing and collaborative working between pharmacological and non-pharmacological treatments, including rTMS, tDCS, cognitive training, and exercise and non-exercise physical activities to introduce appropriate support according to changes in functional needs and prevention or treatment of vascular risk factors. An effective multimodal intervention process makes it possible to improve, sustain, and even delay the decline in overall functional performance of people with PSCI or VCI. The assessment of daily occupations should be further evolved to provide detailed information about the deficits in activities of daily living and consequently improve treatment interventions.

### What development process should be proposed?

**Fig. 5:**
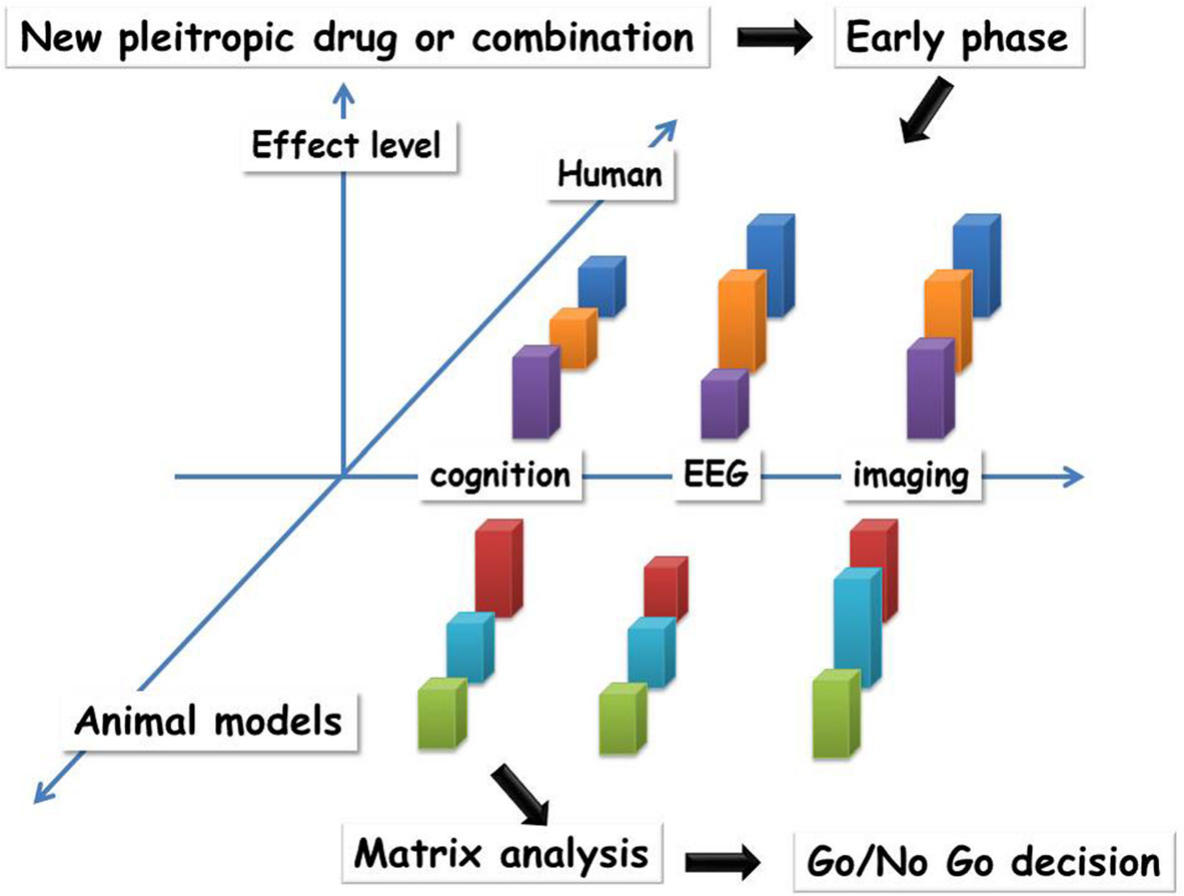
Early phases of strategy development through a matrix approach using a biomarker battery. The aim of this process is to reinforce the rationale of Go/No Go decision before the translation to phase 3. For each animal or clinical study, the effect level should be determined and integrated into a matrix model in order to properly assess the relevance of the tested strategy

In the near future, the main challenge will be to propose a way to develop new or repurposing drugs, alone or in combination with either a pharmacological or non-pharmacological approach, in PSCI or VCI. The crucial step of this challenge will take place in preclinical or clinical early phases before Go/No Go decision to continue into phase 3. At this stage, a matrix approach should be used, including multiple animal models, healthy volunteers, and a stratified patient population, with several sources of assessment through different clinical, neurophysiological, or imaging biomarkers (Fig. 5). A specific overview of the types of failures in clinical trials can be found in the World Federation of Societies of Biological Psychiatry guidelines on dementia treatment, with an emphasis on the failures that are preventable [103]. In this context, a group of researchers has recently published a guideline to support investigators in developing the design of studies; they have identified several issues to be addressed, namely clarifying the research question, describing dropouts and survival, selecting appropriate study participation, practice effects, reliability, and unequal interval scaling, specifying the time scale, the non-linearity development of the severity of cognitive symptom, the time varying exposures and confounding, and the false discovery and over-fitting [104].

The choice of tested strategies should be driven by pathophysiological targets with relevant combinations to exert pleiotropic and multimodal effects. Preparing this choice will start with a systematic literature search and the definition of a preclinical and clinical study design. When a literature search is performed, caution has to be taken to avoid misleading conclusions. For meta-analyses and reviews, Rosenthal and De Matteo [105] outlined the advantages and disadvantages. Moreover, it should be kept in mind that the peer-review system does not always guarantee scientific excellence [106]. Moreover, to prove the details of studies, reviews and meta-analyses are not always useful.

**Fig. 6:**
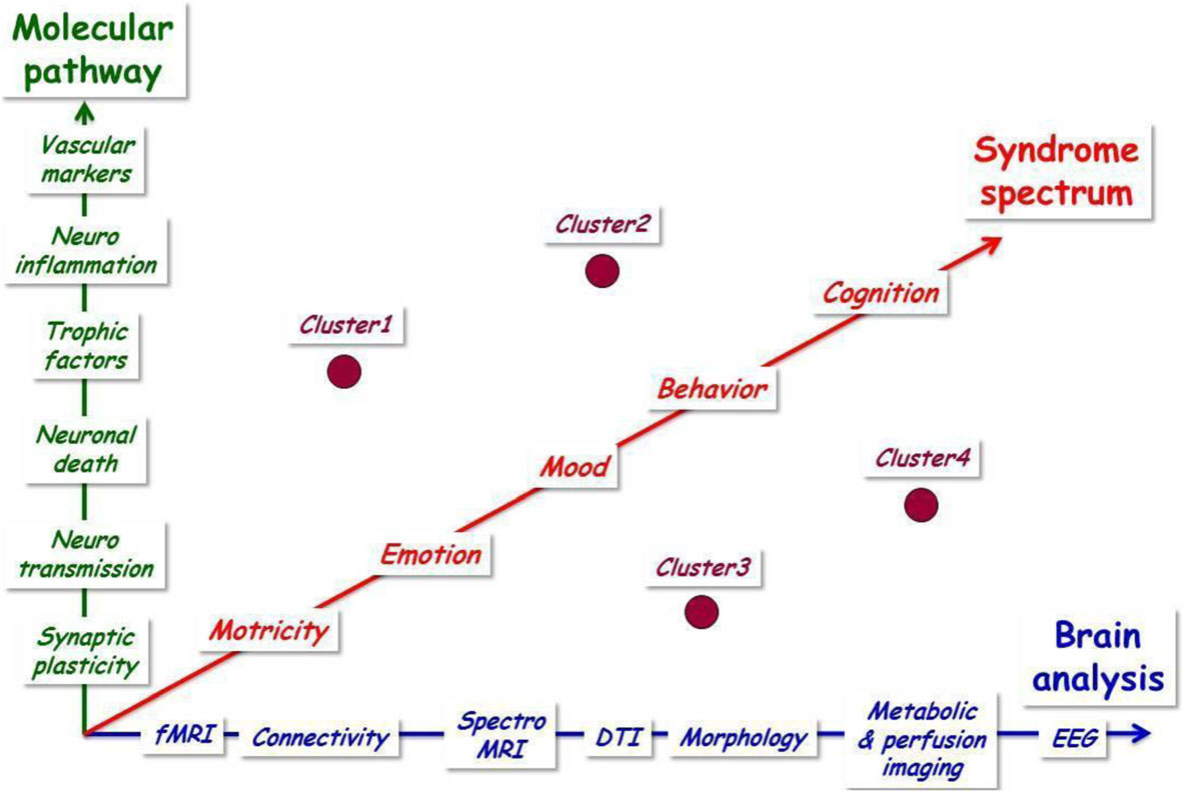
Multiple biomarkers for patient clustering within the spectrum of vascular dementia. Clinical, biological, functional, and lesion biomarkers should be integrated in an algorithm to identify relevant and homogenous patient clusters, with a view to a more accurate predictive diagnosis and stratification for inclusion in clinical trials. *fMRI* functional magnetic resonance imaging, *DTI* diffusion tensor imaging, *EEG* electroencephalography

Valid study designs are needed for both pharmacological and non-pharmacological treatment of PSCI or VCI, by defining basic methodological issues, such as the instruments that should be used to measure eventual changes, the number of participants to investigate, the statistical tests to be used, and the inclusion and exclusion criteria to be defined. Moreover, the experimental design should also be addressed; the standard design is a randomized, double blind, placebo-controlled study that aims to demonstrate improvement in at least one symptom, although this is not always considered the best solution, in particular because of the natural history of the disease or ethical reasons [107]. An answer to this question might come from statistics, by calculating the number needed to treat [108] or the real probability of an outcome. This underlines the need of including adequate statistical knowledge in the preparation of a study design. Additionally, special challenges have to be taken into account, such as the different possible diagnostic definitions of VaD spectrum, the co-morbid diseases, and the high number of possible intervening variables. Here, an overview of possible pitfalls when creating study designs has been provided and conclusions on more improved designs derived. Investigations in VaD bear a higher potential of methodological pitfalls than studies in most of other dementias since both the causes and course of VaD are heterogeneous and, in most cases, not predictable, leading to a high variability in what needs to be investigated. This heterogeneity means the need to develop clinical, biological, functional, and lesion biomarkers to identify relevant and homogenous patient clusters, with a view to a more accurate predictive diagnosis and stratification for inclusion in clinical trials (Fig. 6). This heterogeneity also explains the need to use several animal models, since only one model is unable to mimic the entire clinical spectrum. Animal models should also combine both specific brain lesions and systemic vascular or metabolic disease.

The design of the preclinical and clinical studies also depends on the outcome. The integration of biomarkers from magnetic resonance imaging (structural and functional, diffusion tensor imaging), electroencephalography, and neurophysiological markers is probably the best way to improve the assessment of drug development for the early phases [109, 110]. Overall, these biomarkers are sensitive to both degenerative and vascular processes, allowing a more precise evaluation of brain functioning in a context of vascular impairment, where cognitive assessment remains largely unspecific. In this regard, electroencephalography cartography and multimodal analysis and sourcing of cerebral rhythms are likely to be a feasible approach to explore changes induced by hypoxia and vascular lesions in both animal models and patients. The use of these biomarkers in phase 2 might be a way to a more rapid identification of the disease-modifying strategies that should be successful in phase 3, with a stop in the process for drugs that only have an elusive impact on these biomarkers.

## Conclusion

For the first time, a scientific and methodological rationale is proposed to highlight the interest of disease-modifying strategy applications in VCI and PSCI. This proposal is based on the observation of pathophysiological substrates and clinical aspects, which are dynamic processes justifying the need of disease-modifying strategies. The second line of conclusion is that this strategy needs to be multimodal to achieve the best chance of success. Indeed, the complexity of pathophysiology explains that modulation of several targets is necessary through pleiotropic drugs or a combination with an emphasis on existing or repurposing drugs. The multimodal approach should be based on both pharmacological and non-pharmacological strategies. Finally, a specific method for the development of such an approach, in particular in the early phases, should associate (1) a better stratification of patients based on several animal models able to mimic the complete spectrum and (2) the integration of biomarkers as outcome measures to refine the assessment of relevant disease-modifying strategies.
